# Dynamics of Two Picophytoplankton Groups in Mediterranean Sea: Analysis of the Deep Chlorophyll Maximum by a Stochastic Advection-Reaction-Diffusion Model

**DOI:** 10.1371/journal.pone.0066765

**Published:** 2013-06-24

**Authors:** Giovanni Denaro, Davide Valenti, Bernardo Spagnolo, Gualtiero Basilone, Salvatore Mazzola, Salem W. Zgozi, Salvatore Aronica, Angelo Bonanno

**Affiliations:** 1 Dipartimento di Fisica e Chimica, Università di Palermo, Group of Interdisciplinary Physics and Consorzio Nazionale Interuniversitario per le Scienze Fisiche della Materia, Unità di Palermo, Palermo, Italy; 2 Istituto per l’Ambiente Marino Costiero, Centro Nazionale delle Ricerche, Unità Operativa di Supporto di Capo Granitola, Campobello di Mazara, Trapani, Italy; 3 Marine Biology Research Centre, Tajura, Libya; National Research & Technology Council, Argentina

## Abstract

A stochastic advection-reaction-diffusion model with terms of multiplicative white Gaussian noise, valid for weakly mixed waters, is studied to obtain the vertical stationary spatial distributions of two groups of picophytoplankton, i.e., picoeukaryotes and Prochlorococcus, which account about for 60% of total chlorophyll on average in Mediterranean Sea. By numerically solving the equations of the model, we analyze the one-dimensional spatio-temporal dynamics of the total picophytoplankton biomass and nutrient concentration along the water column at different depths. In particular, we integrate the equations over a time interval long enough, obtaining the steady spatial distributions for the cell concentrations of the two picophytoplankton groups. The results are converted into *chlorophyll a* and *divinil chlorophyll a* concentrations and compared with experimental data collected in two different sites of the Sicily Channel (southern Mediterranean Sea). The comparison shows that real distributions are well reproduced by theoretical profiles. Specifically, position, shape and magnitude of the theoretical deep chlorophyll maximum exhibit a good agreement with the experimental values.

## Introduction

The study of vertical profiles of phytoplankton in marine ecosystem is of fundamental importance to know the dynamics and structure of aquatic microorganisms [1]–[6]. In previous works, the distribution of phytoplankton in oceans and lakes have been obtained by using a deterministic approach to describe and reproduce the experimental data for the chlorophyll concentration. Two novelties are present in this work: i) the use of a stochastic approach to model the dynamics of more phytoplankton populations; ii) the comparison between theoretical and experimental distributions of chlorophyll concentration; this is performed by using, for each phytoplankton population, a conversion curve to obtain from the biomass concentrations the equivalent chlorophyll content. It is important to stress that marine ecosystems, because of the presence as well of non-linear interactions among their parts as deterministic and random perturbations due to environmental variables, are complex systems [7]–[23]. Therefore, in order to better reproduce this non-linear and noisy dynamics, it is necessary that the model takes into account the presence of external random fluctuations [24], [25] including, in the equations of our model, terms of multiplicative noise [14], [26]–[28].

Phytoplankton is an essential component of all aquatic ecosystems in terms of biomass, diversity and production [29], [30], and is responsible for a significant fraction of marine primary production [31], [32]. The phytoplankton communities and their abundances depend on several phenomena of hydrological and biological origin, and involve different limiting factors [33]. The Mediterranean waters are generally characterized by oligotrophic conditions, and a previous work [34] has suggested that there is a decreasing trend over time in chlorophyll concentration in the Sicily Channel. This has been associated with increased nutrient limitation resulting from reduced vertical mixing due to a more stable stratification of the basin, in line with the general warming of the Mediterranean Sea [34]–[36].

It is worth noting that the production of fish species depends on the primary production of phytoplankton [30], [37]–[39]. In general, the variations in the anchovy growth among different areas are mainly explained by changes in the chlorophyll concentration. In particular, due to a decrease of biomass concentration in last years, we observed that the values of the anchovy growth in some regions of the Sicily Channel result to be in the low end of the range [40]. Therefore, this limited fish production could be a marker of low phytoplankton concentration, indicating a weak primary production in this area [59], [68].

In this work we report on a study conducted in a hydrologically stable area of Mediterranean Sea, where the light intensity and nutrient concentration select different species and groups along water column, contributing to determine the biodiversity of the ecosystem. In fact, the growth of all phytoplankton groups is limited by the intensity of light 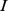 and concentration of nutrients 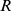 [2], [5], [6], [41]. The light penetrates through the surface of the water and decreases exponentially along the water column. The nutrients, which are in solution, come from deeper layers of water column, near the seabed, and are characterized by an increasing trend from the surface waters to the benthic layer [2]–[4], [28]. In Sicily Channel phosphorus, which is contained in phosphates present in solution, is the nutrient component playing the role of limiting factor for the growth of phytoplankton groups [42], [43].

The responses of each picophytoplankton species to environment solicitations strongly depend on the biological and physical parameters [5], [44], [45]. In particular, half-saturation constants determine the position of the production layer and depth of concentration peak for every aquatic microorganism species, while the sinking velocity is strictly connected with the phytoplankton size. Moreover, it is known that growth rates and nutrient uptake play a main role in the balance of a marine ecosystem [6], [46], [47], and contribute to modify the composition of the phytoplankton communities.

In this paper we investigate two sites of the Strait of Sicily, localized between the eastern and western basins of Mediterranean Sea and characterized by weakly mixed water. The purpose of this work is to simulate the spatio-temporal distributions of two groups of picophytoplankton, i.e. picoeukaryotes and Prochlorococcus, which account about for 60% of total *chl a* and *Dvchl a* concentration on average in Mediterranean Sea [48], [49]. In order to study our marine ecosystem, it is necessary to set the correct values of the parameters. These have to guarantee the coexistence of the two groups [4], [50], i.e. picoeukaryotes and Prochlorococcus, in the deep chlorophyll maximum (DCM).

Initially we use a deterministic advection-reaction-diffusion model to analyze the spatio-temporal evolution of the biomass concentrations of both groups, obtaining the distributions of the total *chlorophyll a* (*chl a*) and *divinil chlorophyll a* (*Dvchl a*) concentrations in stationary regime. Afterwards, in order to take into account the randomly fluctuating behaviour of the environmental variables, we study the ecosystem dynamics by a stochastic approach, by inserting terms of multiplicative Gaussian noise in the system equations. The numerical results are compared with experimental data sampled in two different sites of the Sicily Channel.

### Environmental Data

**Figure 1:**
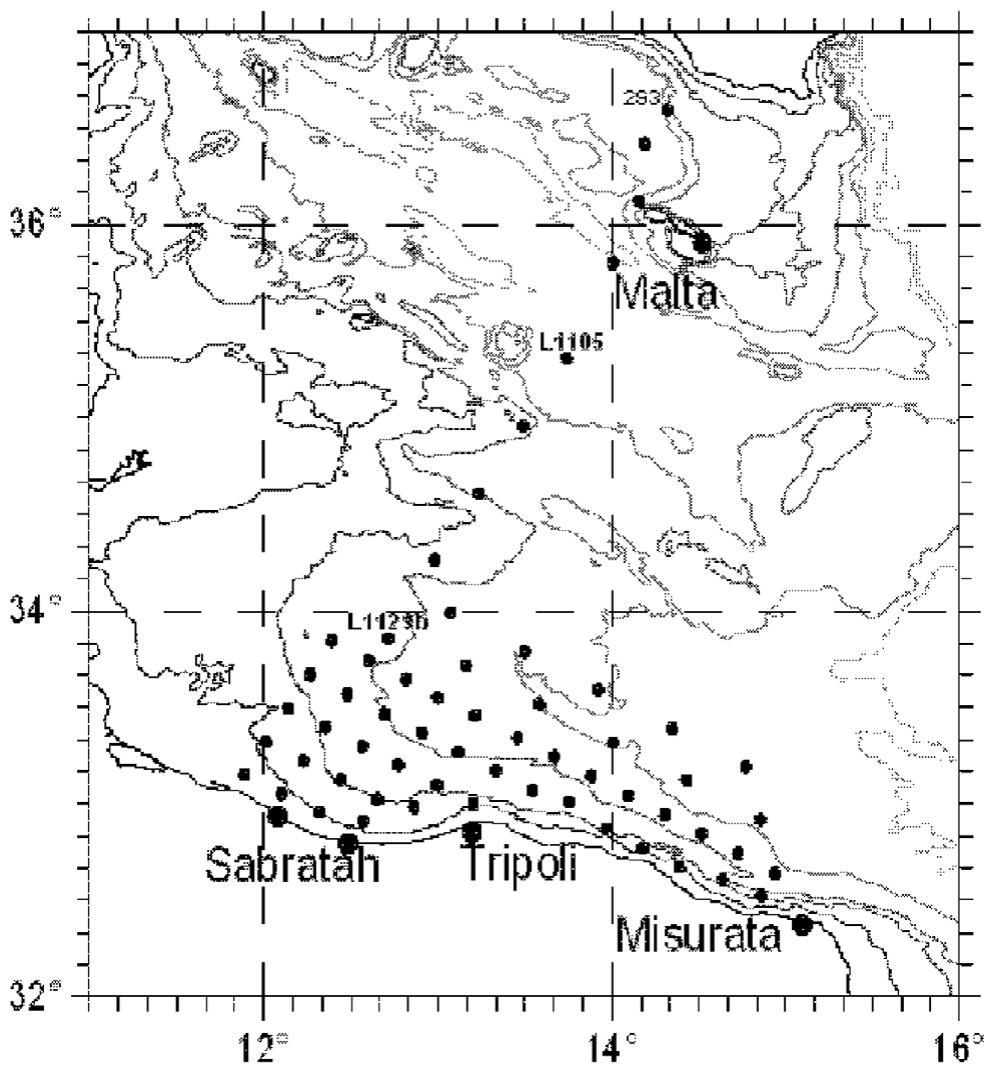
Locations of the CTD stations where the experimental data were collected. (Courtesy of Valenti et al., 2012 (Ref. [27])).

Data used in this work were acquired in the period 12
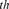
–24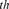 August 2006 in the Sicily Channel area (Fig. 1) during the MedSudMed-06 Oceanographic Survey onboard the R/V Urania. Conductivity, temperature and density were sampled by means of the SBE911 plus CTD probe (Sea-Bird Inc.), while *chlorophyll a* and *divinil chlorophyll a* fluorescence measurements (*chl a*/*divinil chl a*, 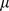
g 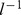) were contemporary performed using the Chelsea Aqua 3 sensor. The CTD stations were located on a grid of 12×12 nautical miles in Mediterranean Sea, and the values of oceanographic parameters were collected along a transect between the Sicilian and the Libyan coasts. The collected data were processed, generating a text file for each station, where the values of the experimental data were estimated with a 1 m step.

In this work, two sites out of the whole data set were considered. In particular, the selected stations were located at south of Malta (site L1105) and on the Libyan continental shelf (site L1129b) (see Fig. 1). Here, hydrological parameters remained constant for the entire sampling period and were representative of the oligotrophic Mediterranean Sea in summer [34]. Nutrient concentrations, i.e nitrate and phosphate, were not sampled.

### Phytoplanktonic Data

The quantity that indicates the presence of phytoplankton biomass in marine environment is the concentration of *chlorophyll a* and *divinil chlorophyll a* [48], [51]. Moreover, the contribution of each phytoplankton group to the total phytoplankton biomass is obtained using group-specific conversion factors empirically determined, and based on the analysis of taxonomic pigments [52], [53]. These pigments have been used as size class markers of two main size fractions: picophytoplankton (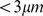) and nano- and micro-phytoplankton (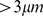).

The picophytoplankton size fraction accounts for 80% of the total *chl a* and *Dvchl a* on average in the Strait of Sicily, and is mainly represented by two groups: picoprokaryotes and picoeukaryotes. The picoprokaryotes group is dominated by two species of cyanobacteria, i.e. Synechococcus and Prochlorococcus, while picoeukaryotes group is mainly represented by pelagophytes and prymnesiophytes [48]. Prochlorococcus, Synechococcus and picoeukaryotes are usually identified and measured based upon their scattering and autofluorescence [48]. This is due to the presence of *chl a* or *Dvchl a* molecules in their cells. Finally, Prochlorococcus and picoeukaryotes contribute equally to the picophytoplankton in terms of *chl a* and *Dvchl a* concentrations, even if Prochlorococcus are numerically more abundant than the picoeukaryotes group [51].

The nano- and micro-phytoplankton fraction amounts in average to 20% of the total *chl a* and *Dvchl a* and is uniformly distributed along the water column. This size fraction is dominated by prymnesiophytes and diatoms.

Picophytoplankton groups present eco-physiological properties [54]–[57] that make them appropriate to be studied by the use of biological models. In fact, the small size of Prochlorococcus and picoeukaryotes leads to a low package effect, which contributes to the light-saturated rate of photosynthesis that can be achieved at relatively low irradiances [54], [58]–[60]. This feature allows the growth of picoeukaryotes in deeper layers of the water column. Conversely, a low nutrient uptake of picoeukaryotes leads to an enough high nutrient concentration in shallower layers of the water column, where Prochlorococcus are localized and their growth is allowed. Because of their peculiarities and relevant role in the functioning of the ecosystem, Prochlorococcus and picoeukaryotes constitute two populations that can coexist in same marine environment. In these conditions they are suitable to be described by a model of population dynamics [3], [4], [50].

**Figure 2:**
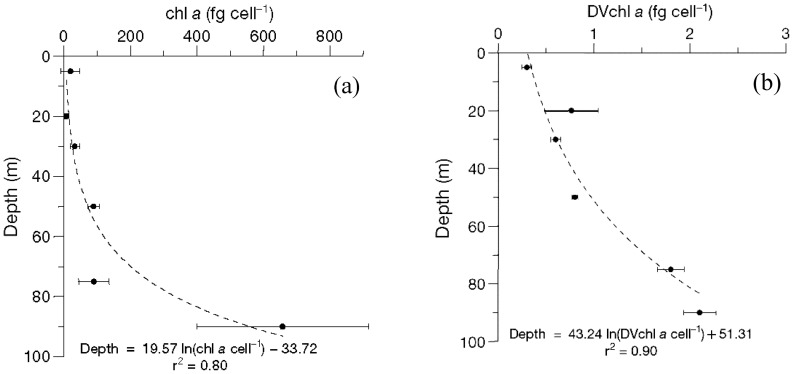
Mean vertical profile of *chl a* per picoeukaryote cell (panel a) and *Dvchl a* per Prochlorococcus cell (panel b). Error bars are Standard Deviation. Equation and 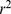 for the fit are reported on the plots. (Courtesy of Brunet et al., 2007 (Ref. [51])).

In the Strait of Sicily [48], [51], [53], the average picoeukaryotes concentration in the DCM is 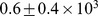 cell ml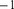, and the mean value of *chl a* cell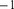 ranges between 10 and 660 fg *chl a* cell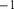 along the water column, with a significant exponential increase with depth (see Fig. 2a) [5]. The concentration of *chl a* per cell in picoeukaryotes is highly variable among different water masses, with significantly higher values in the DCM respect to the surface [55], [59], [61], [62].

In our ecosystem, picophytoplankton is numerically dominated by Prochlorococcus with average concentrations of 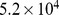 cell ml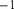. This species is more concentrated in DCM, where can achieve the mean value of 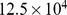 cell ml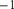. In particular, the marker of Prochlorococcus is *divinil chlorophyll a*, whose molecular structure is almost identical to that of *chlorophyll a*. The *Dvchl a* cellular content of total Prochlorococcus ranges between 0.25 and 2.20 fg *Dvchl a* cell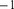 along the water column, with a mean value exponentially increasing with depth (see Fig. 2b) [51].

Experimental analysis performed on samples collected in Sargasso Sea and Mediterranean Sea showed that the cellular content of *chl a* and *Dvchl a* increases in Prochlorococcus and Synechococcus with decreasing light intensity [63]. In particular, ranging from 1500 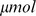 photons 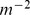 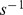 near the surface to less than 1 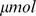 photons 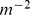 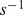 below the euphotic zone (approximately 100 m in Mediterranean Sea during the summer period), cells display a variety of differences. The most obvious ones are concomitant increases in cell size and pigment content, which generally occur below the depth of the mixed layers [64]. On the other side, for depth greater than 100 m, the cell concentration of picophytoplankton shows a considerable decrease, due to the dramatic diminution of the light intensity, which becomes less than 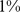 of the light intensity at the sea surface. The consequent strong reduction of cell concentration below euphotic zone allows to exploit the conversion curves shown in Fig. 2 also for depth below 100 m, describing, without significative errors, the increase in pigment content per cell.

In general, picophytoplankton ranges from 40% to 90% of total *chl a* along the water column, with an average value of 69% close to the DCM. Picoprokaryotes are dominant in the first 50 meters, but are replaced by picoeukaryotes in deeper water [51].

**Figure 3:**
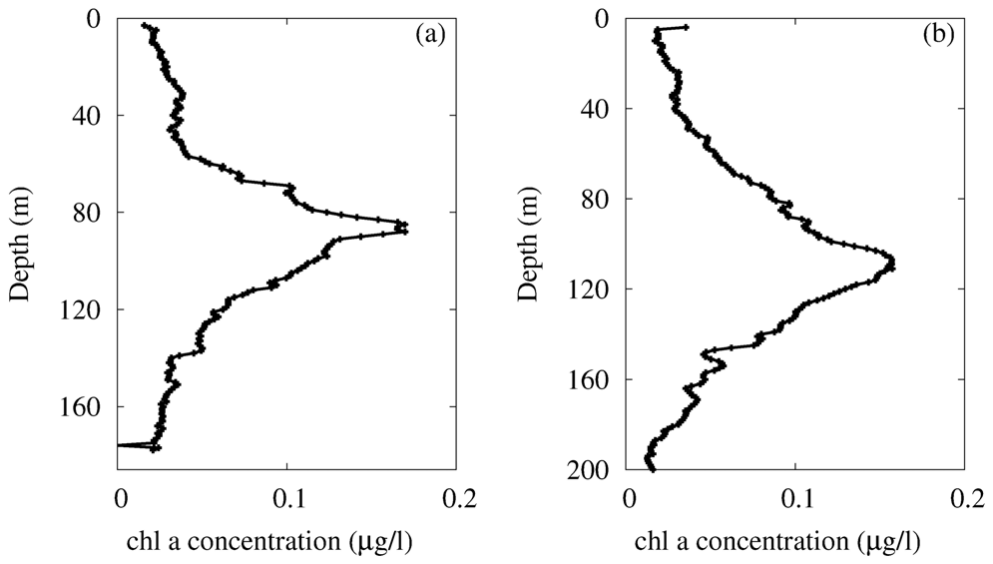
Profiles of *chl a* concentration measured in sites L1129b (panel a) and L1105 (panel b). The black lines have been obtained by connecting the experimental points corresponding to samples distanced of 1 meter along the water column. The total number of samples measured in the two sites is 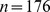 for L1129b, and 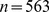 for L1105. (Courtesy of Denaro et al., 2013 (Ref. [28])).

The fluorescence profiles for *chl a* concentration, acquired in the Sicily Channel during the MedSudMed-06 Oceanographic Survey, show a nonmonotonic behaviour, as a function of the depth, characterized by the presence of DCM in both sites (see Fig. 3). In particular, the *chlorophyll a* concentrations range between 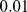 and 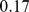 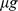 *chl a* l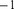, with a deep chlorophyll maximum always present between 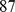 and 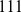 m depth. Finally, we observed different depth, shape and width of the DCM in the two sites studied.

## Methods

**Figure 4:**
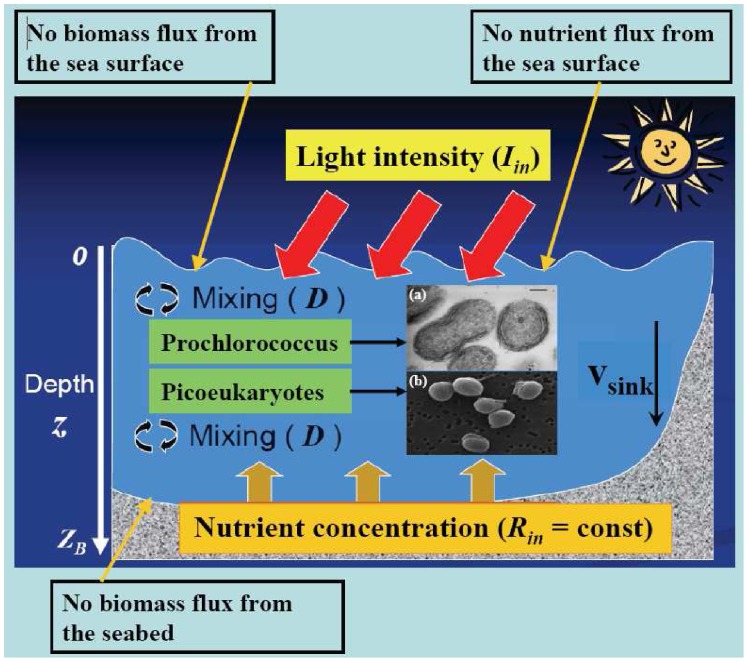
Scheme of the mechanism responsible for the phytoplankton dynamics (modified from original figure by Alexey Ryabov). Inset: (a) Prochlorococcus PCC 9511 (courtesy of Rippka et al., 2000 (Ref. [99])), (b) Micromonas NOUM17 (courtesy of Augustin Engman, Rory Welsh, and Alexandra Worden). (Color online).

The spatio-temporal behaviour of the two picophytoplankton groups is analyzed by using a stochastic model with conditions typical of the Mediterranean Sea, where the vertical water columns are weakly mixed. In Fig. 4 we give a schematic representation of the mechanism underlying the phytoplankton dynamics. The mathematical tool used to simulate the picophytoplankton dynamics is an advection-reaction-diffusion model. The analysis is performed considering two different populations: (i) picoeukaryotes group; (ii) Prochlorococcus, which is a species belonging to the picoprokaryotes group. Analysis and numerical elaborations are divided in two phases:

- *Phase 1*. The distributions of biomass concentrations 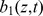 (picoeukariotes) and 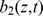 (Prochlorococcus), and nutrient concentration 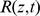 are obtained along the weakly mixed water column as a function of the time *t* and depth *z*, by using a deterministic model based on three differential equations. The results obtained are in a good qualitative agreement with the experimental data collected in the two different sites of the Strait of Sicily.
- *Phase 2*. In order to obtain, from a quantitative point of view, a more significative agreement between theoretical results and experimental data, the random fluctuations of the environmental variables are taken into account. In particular, a stochastic model is devised, starting from the deterministic one and inserting into the equations terms of multiplicative Gaussian noise. Specifically, as a first step, a term of multiplicative noise is added only in the differential equation for the nutrient concentration (case 1). As a second step, terms of multiplicative noise are inserted also into the equations for the biomass concentrations of picoeukariotes and Prochlorococcus (case 2). By this way, the effects of the environmental noise on picophytoplankton distributions are analyzed.

### The Deterministic Model

In this section, we consider a deterministic advection-reaction-diffusion model [2]–[4] to analyze the dynamics of the two picophytoplanktonic groups, distributed along a one-dimensional spatial domain (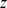-direction). In particular, we assume that the interaction of these populations with the environment occurs through two factors that limit the growth of the aquatic microorganisms: light intensity and nutrient, i.e. phosphorus. The model allows to obtain the dynamics of the biomass concentrations of picoeukaryotes and Prochlorococcus, 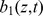 and 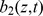, nutrient concentration 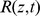 and light intensity 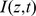. A crucial role in the phytoplankton dynamics is played by three different factors: growth and loss of biomass concentration, and movement of the single microorganisms.

The growth rates of the two picophytoplankton groups are strictly connected with 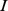 and 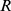, whose characteristics of limiting factors [2], [33], [41], [65] are implemented in the model by the Monod kinetics [66]. The gross phytoplankton growth rates per capita are given by 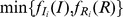, where 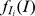 and 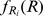 are obtained by the Michaelis-Menten formulas
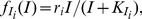
(1)

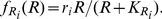
(2)where 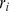 is the maximum growth rate, and 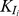 and 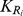 are the half-saturation constants for light intensity and nutrient concentration, respectively, of the *i-th* picophytoplankton group. These constants depend on the metabolism of the specific microorganisms considered. In particular, 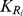 and 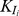 contribute to determine the position along the water column (depth) of the maximum (peak) of biomass concentration for each species. The biomass loss of the *i-th* picophytoplankton group, connected with respiration, death, and grazing, occurs at a rate 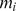 [2]–[4]. The gross per capita growth rates are defined as



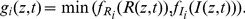
(3)The movement of phytoplankton groups depends on turbulence, responsible for a passive movement of the phytoplankton. Turbulence is modeled by vertical diffusion coefficient 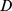, which we assume uniform with the depth in both sites. Sinking velocities of the two picophytoplankton groups, 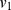 and 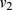, describe another passive movement of picoeukaryotes and picoprokaryotes along water column towards deeper layers [2], [50], [67]. Positive velocities are oriented downward (sinking) for both groups, and are set equal to those observed in experimental data [3], [4].

Taken together, these assumptions about growth, loss, and movement result in the following differential equations for the dynamics of the biomass concentrations of picoeukaryotes 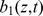 and Prochlorococcus 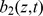 [3], [4], [50].
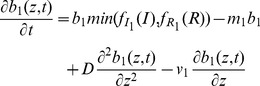
(4)

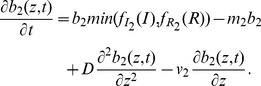
(5)


Boundary conditions for concentrations of picoeukaryotes and Prochlorococcus biomass describe no-flux in both surface layer 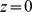 and seabed 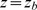:
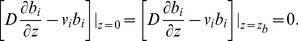
(6)


The nutrient concentration 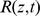 is consumed by both the picophytoplankton groups, and a further quantity of nutrient is obtained from dead phytoplankton by a recycling process. Furthermore, turbulence is also responsible for mixing of the nutrient concentration along the water column and it is described by the vertical diffusion coefficient 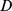. All these processes are modeled by the following equation.
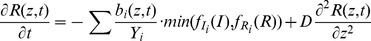


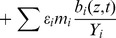
(7)where 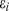 and 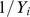 are nutrient recycling coefficient and nutrient content of the *i-th* picophytoplankton group, respectively.

Nutrients do not come from the top of the water column but are supplied from the bottom. In particular, nutrient concentration at the bottom of the water column, 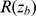, is fixed at value 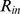, which is different in the two sites investigated. Thus the boundary conditions are described by the following equations:
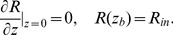
(8)


The light intensity is assumed to decrease exponentially according to Lamber-Beer’s law [5], [68], [69].
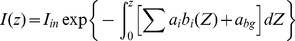
(9)where 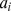 are the absorption coefficients of the *i-th* picophytoplankton group, 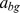 is the background turbidity, and 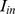 is the incident light intensity at the water surface.

### The Stochastic Model

The theoretical model discussed in the previous section is based on a deterministic approach. However, it is worth to recall that the marine ecosystems are complex systems, that is open systems characterized by non-linear interactions [14], [25], [28], [70]–[77]. In particular, each picophytoplankton group not only interacts with all other populations, but is also subject to environmental variables, such as turbulence and availability of food resources, which affects the ecosystem dynamics through deterministic and random perturbations. In this context, random variations of species concentrations [7]–[9], [18], [21] are fundamental aspects that can not be neglected when seeking a better understanding of the dynamics of complex living systems. Here the fluctuations of temperature, food resources, and other environmental parameters can be modeled by including multiplicative noise sources [10], [11], [14], [70], that can effectively reproduce experimental data in population dynamics [78]–[80].

We note that the same arguments hold for nutrients, whose random fluctuations should be modeled by terms of multiplicative noise, according to the approach widely used to describe stochastic dynamics not only in physics, but also in biology, ecology, economy, or social sciences [79]. This agrees with the observation that the effects of fluctuations have to be proportional to the activity densities [81]–[85], which are in our system the biomass and nutrient concentrations.

We recall also that problems, which involve absorbing states, are described by equations whose noise amplitude is proportional to the square root of the space and time dependent activity density. Such systems include propagating epidemics, autocatalytic reactions, and reaction-diffusion problems [79].

Finally we underline that in our ecosystem biomass and nutrient concentrations are affected by unpredictable changes mainly generated by two sources of fluctuations: i) vertical mixing along the water column due to the random variations of the velocity field, ii) gain or loss of biomass and nutrient concentrations among different water columns due to random horizontal movement. Thus the multiplicative noise, used in population dynamics and reaction-diffusion problems [78], [86]–[88], within our specific physical and biological context describes the two above mentioned noise sources. These are responsible for the real behaviour of the ecosystem, characterized by an intrinsically non-deterministic dynamics.

Therefore, in order to reproduce the dynamics of the picophytoplankton groups and nutrient concentration, taking into account the role of environmental fluctuations, we modify the model given by Eqs. (4)–(9), including terms of multiplicative noise.

**Case 1.** The environmental noise affects only the nutrient concentration. In this case, Eqs. (4),(5),(6),(8),(9) remain unchanged, while Eq. (7) is replaced by
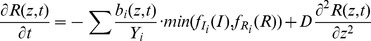


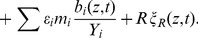
(10)


**Case 2.** The environmental noise affects the concentrations of picoeukaryotes biomass, Prochlorococcus biomass and nutrient. Therefore, Eqs. (6),(8),(9) are the same, while Eqs. (4),(5) and (7) become.
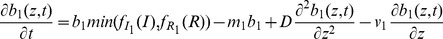


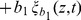
(11)

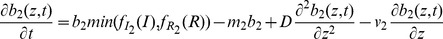


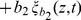
(12)

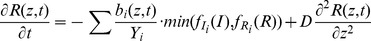


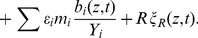
(13)


Here 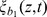, 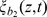 and 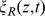 are statically independent and spatially uncorrelated white Gaussian noises with the following properties: 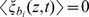, 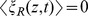 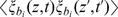 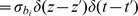, 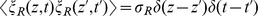, with 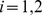. Here 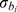 and 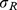 are the intensities of the noise sources which act on the *i-th* picophytoplanktonic group and nutrient, respectively.

### Simulation Setting

In order to reproduce the spatial distributions observed in the experimental data for the total concentration of *chl a* and *Dvchl a* (see Fig. 3), we choose the values of the environmental and biological parameters so that the monostability condition is obtained. This corresponds to the presence of a deep chlorophyll maximum for both picophytoplankton groups [2]–[4], [28], which coexist in the same ecosystem even if the maximum concentration for each group is localized at a different depth [5]. The numerical values assigned to the parameters are shown in Table 1.

**Table 1:** Parameters used in the model.

Symbol	Quantity	Unit	Site L1129b	Site L1105
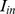	Incident light intensity	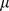 mol photons m 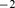 s 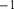	1404.44	1383.19
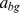	Background turbidity	m 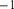	0.045	0.045
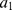	Absorption coefficient of picoeukaryotes	m 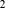 cell 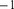	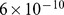	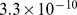
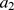	Absorption coefficient of Prochlorococcus	m 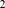 cell 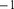	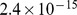	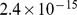
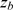	Depth of the water column	m	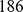	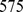
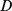	Vertical turbulent diffusivity	cm 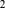 s 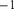	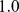	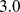
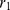	Maximum specific growth rate of picoeukaryotes	h 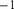	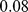	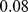
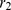	Maximum specific growth rate of Prochlorococcus	h 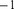	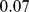	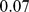
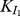	Half-saturation constant of light-limited growth of picoeukaryotes	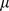 mol photons m 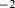 s 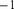	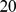	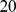
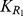	Half-saturation constant of nutrient-limited growth of picoeukaryotes	mmol nutrient m 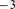	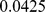	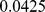
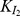	Half-saturation constant of light-limited growth of Prochlorococcus	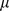 mol photons m 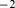 s 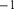	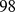	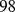
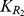	Half-saturation constant of nutrient-limited growth of Prochlorococcus	mmol nutrient m 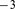	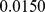	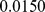
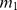	Specific loss rate of picoeukaryotes	h 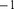	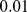	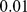
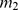	Specific loss rate of Prochlorococcus	h 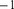	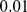	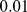
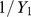	Nutrient content of picoeukaryotes	mmol nutrient cell 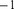	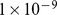	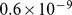
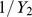	Nutrient content of Prochlorococcus	mmol nutrient cell 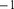	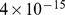	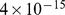
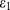	Nutrient recycling coefficient of picoeukaryotes	dimensionless	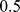	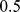
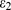	Nutrient recycling coefficient of Prochlorococcus	dimensionless	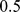	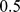
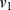	Sinking velocity of picoeukaryotes	m h 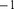	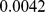	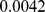
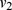	Sinking velocity of Prochlorococcus	m h 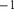	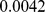	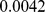
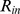	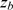 Nutrient concentration at	mmol nutrient m 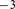	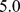	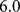

The values of the biological parameters are those typical of picoeukaryotes and Prochlorococcus.

The values of the biological parameters have been chosen to reproduce the behaviour of picoeukaryotes and Prochlorococcus. In particular, for both groups, the maximum specific growth rates are in agreement with ones measured from other authors [89] and the sinking velocity is set to the typical value for picophytoplankton, 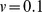 m day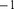 [3]. The half-saturation constants, 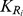 and 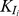, for the two groups are set to obtain a suitable position of production layers and a certain depth for the position of the peak of biomass concentration. Since picoeukariotes consist of picophytoplankton species that are better adapted to lower light intensity than Prochlorococcus, we fix 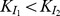. Viceversa, since Prochlorococcus is better adapted to lower nutrient concentration than picoeukariotes group, we set 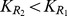. As a consequence, the peak of picoeukaryotes concentration along the water column tends to be deeper than the peak of Prochlorococcus concentration. It is worth noting that the nutrient content of the picoeukaryotes, 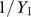, is set to different values in the two sites investigated in this work. This choice can be explained recalling that, in the Mediterranean Sea, the picoeukaryotes group located in DCM includes several species. As a consequence, depending of the marine site analyzed, different ecotypes of this group prevail and nutrient content changes accordingly [6], [90]. Viceversa, the nutrient content of picoprokaryotes (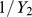) is set equal in both sites because Prochlorococcus is the only species of its group present in DCM. We recall that the parameters 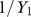 and 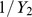 contribute to determine the steady distributions of the picophytoplankton concentrations. Experimental findings indicate that (i) the peak of biomass concentration of Prochlorococcus is shallower than that of picoeukaryotes and (ii) the cell concentration of Prochlorococcus is much higher than that of picoeukaryotes. In these conditions a smaller amount of nutrient is available for Prochlorococcus localized in the biomass peak. Therefore, in order to obtain for the two picophytoplankton groups, the correct cell concentrations as found in field observations, 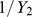 is set at a value much smaller than 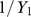 (see Table 1). The absorption coefficient of Prochlorococcus, fixed in our model, is very different from that of the picoeukaryotes. In fact, due to the low nutrient concentration in higher layers and different average cell concentration of the two groups (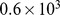 cells ml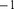 for picoeukariotes and 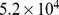 cells ml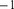 for Prochlorococcus), we had to exploit an absorption coefficient for Prochlorococcus lower than that used for picoeukaryotes. In particular, in order to obtain the same gradient of light intensity inside the production layers [50], we set 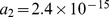
m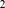 cell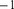. All the other biological parameters are the same in both sites in agreement with ones used from other authors [3], [4].

The values of the environmental parameters have been chosen to reproduce marine ecosystem of Sicily Channel in summer, i.e. oligotrophic water and high light intensity. The water column depths used in the model are fixed according to those measured in the corresponding marine sites. The diffusion coefficients are fixed at typical values of weakly mixed water (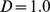 cm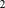 s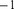 for site L1129b and 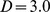 cm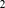 s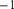 for site L1105). This choice is due to the fact that the site L1129b is placed on the Libyan continental shelf, not far from the coast, where turbulence is low. Conversely, the site L1105 is located in the middle of Sicily Channel, where vertical diffusion coefficient is greater respect to the Libyan coast because the flow of the Modified Atlantic Water (MAW) and Levantine Intermediate Water (LIW) are responsible for a bigger turbulence. Moreover, we set that the light intensity at the water surface, 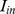, is larger than 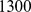 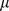
mol photons m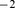 s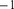 in both sites. This is due to the fact that the sampling of the experimental data occurred during summer (August 2006), when the light intensity achieves maximum values in Mediterranean Sea. In particular, the light intensities used in this study were fixed using data available on the NASA web site (http://eosweb.larc.nasa.gov/sse/RETScreen/). Finally, nutrient concentrations at depth 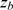 were fixed at values such as to obtain, for each site, a peak of biomass concentration at the same position of the peak experimentally observed.

## Results

The spatio-temporal dynamics of the biomass and nutrient concentrations are obtained by numerically integrating Eqs. (4)–(9). The numerical method, whose computer implementation consists in a C++ program, exploits an explicit finite difference scheme. In order to get the steady spatial distributions, we solved numerically our equations over a time interval long enough to achieve the stationary solution [91]. As initial conditions, for each marine sites analyzed, we fixed that the picoeukaryotes and Prochlorococcus biomasses are concentrated in two layers close to the deep chlorophyll maximum experimentally observed. On the other side, the phosphorus concentration decrease linearly above seabed up to DCM, while remain approximately constant from this point to the water surface.

### Solution by Deterministic Approach

A preliminary analysis (results here not shown) indicates that large values of 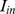 lead to stationary conditions characterized by the presence of a DCM, where two species can coexist, while large values of 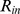 (nutrient concentration close to seabed) determine an upper chlorophyll maximum (UCM) [4], where picoeukaryotes prevail and Prochlorococcus undergoes a strong reduction. In particular, for fixed values of 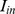 and 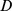, an increase of 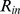 generates a displacement of picoeukaryotes towards higher layers, where the production layer of Prochlorococcus is located. As a consequence of light limitation, Prochlorococcus moves upward in the direction of surface layers of the water column. If 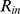 is very high, we can observe an upper chlorophyll maximum (UCM) due to the picoeukaryotes group and the disappearance of Prochlorococcus. These results are in agreement with those shown in Ref. [50].

**Figure 5:**
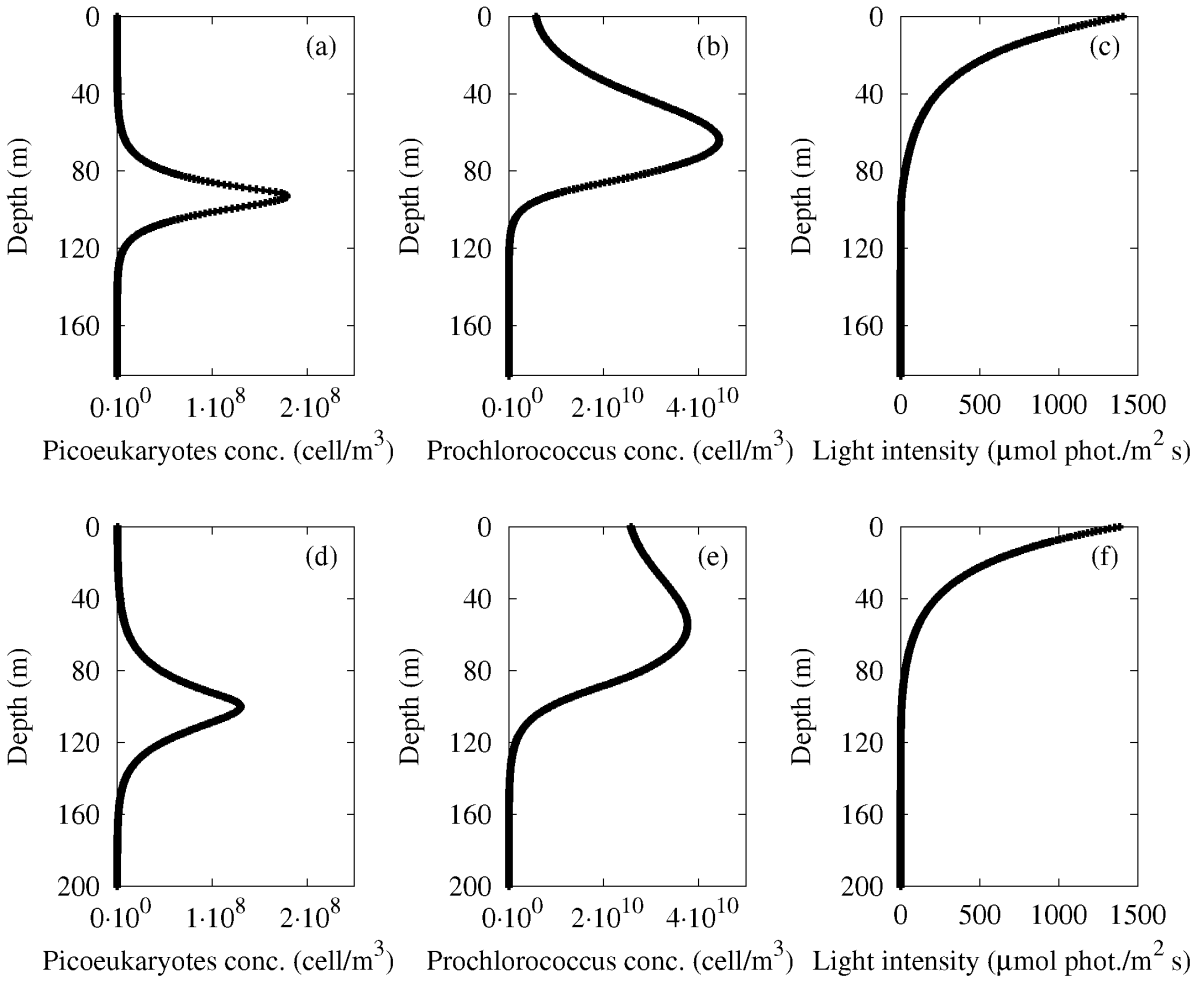
Stationary distributions of picoeukaryotes and Prochlorococcus biomass concentrations and light intensity: site L1129b (panels a, b, c) and site L1105 (panels d, e, f) as a function of depth.

By solving Eqs. (4)–(9) for the maximum simulation time 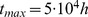, the stationary solution already appears for 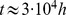. Therefore, in order to obtain the stationary distributions for the biomass concentrations of picoeukaryotes and Prochlorococcus, and the profile of light intensity, it is sufficient to set 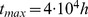. The results are shown in Fig. 5. We observe the presence of a picoeukaryotes biomass peak (panels a, d of Fig. 5) in correspondence of the two experimental DCMs (see Fig. 3). Moreover, a Prochlorococcus biomass peak (panels b, e of Fig. 5) is observed close to the two experimental DCMs (see again Fig. 3). Finally the typical exponential behaviour of the light intensity is found (panels c, f of Fig. 5).

**Figure 6:**
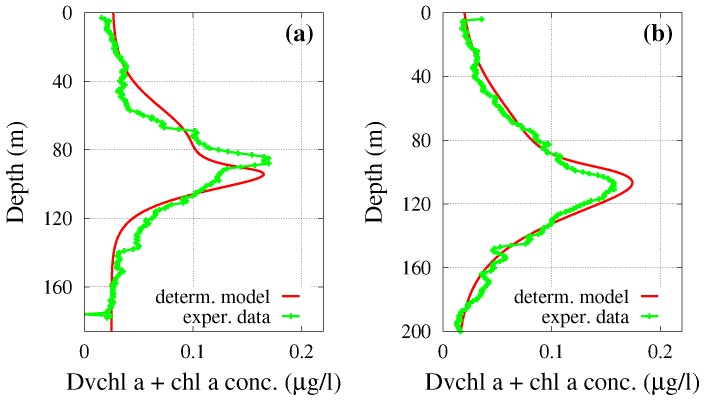
Theoretical distributions (red line) of the total *chl a* and *Dvchl a* concentration in stationary conditions. The profiles, obtained by the deterministic model and given as a function of the depth, are compared with experimental distributions (green line) sampled in sites L1129b (panel a) and L1105 (panel b). (Color online).

We recall that our experimental data are expressed in 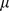
g/l (see Fig. 3), which is the unit of measure used for *chl a* and *Dvchl a* concentrations. Therefore, in order to compare the numerical results with experimental profiles, the theoretical cell concentrations of picoeukaryotes and Prochlorococcus (expressed in cell/m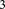) have been converted into *chl a* and *Dvchl a* concentrations (expressed in 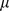
g/l) by using, respectively, the curves of mean vertical profile obtained by Brunet et al. [48], [51]. Since the structure of the *chlorophyll a* molecule is almost identical to that of *Dvchlorophyll a*, we summed their concentrations to get theoretical equilibrium profiles consistent with those obtained from the experimental data. It is also important to recall that in Sicily Channel nano–phytoplankton, micro-phytoplankton and Synechococcus account about for 43% of the total quantity of *chl a* and *Dvchl a* [48], [51]. This quantity is quite uniformly distributed along the water column. Therefore, we considered this fraction of the total biomass and divided it by depth, obtaining for each site the value 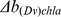, which represents a constant concentration along the whole water column, due to other phytoplankton species present in the marine ecosystem [28]. Then, along the water column, we added the numerical concentrations with 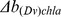 and obtained, for both sites, the stationary theoretical profiles consistent with the experimental ones (see Fig. 6). Here it is possible to observe a fairly good agreement between experimental data (green line) and numerical results (red line). However, in site L1129b the theoretical distribution of *chl a* and *Dvchl a* is characterized by a shape quite different from that of the experimental profile. Moreover, in site L1105 we note that the magnitude of the theoretical DCM is larger than that obtained from the real data. Finally, we performed a quantitative comparison based on the goodness-of-fit test 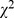. The results (here not shown) indicate that, respect to the one-species model, this description provides in both sites theoretical results in a better agreement with the experimental findings [27], [28].

### Solution by Stochastic Approach

In this section we perform the analysis of the stochastic model by numerically solving the corresponding equations. About the numerical integration, we recall that the calculus of stochastic differential equations with terms of white noise can be based on different definitions, i.e. Ito and Stratonovich schemes. This situation has led to a long controversy in physical literature. In particular, the Stratonovich’s choice is the only definition of stochastic integral leading to a calculus with classic rules within the context of functional analysis. Moreover, a principle of invariance of the equation under “coordinate transformation” is invoked to pick the Stratonovich integral as the “right” one and reject the Ito integral as the “wrong” one. The principle refers to an invariance of the form of the stochastic differential equation under a non-linear transformation of the system. This invariance does not posses any physical virtue, but it is only a different way to say that the Stratonovich calculus obeys the familiar classic rules. The only quantities that have to be invariant under a coordinate transformation are the probabilities. This condition is of course guaranteed in both calculi. Finally we note that in biological applications often environmental fluctuations have a correlation time that is much shorter than the generation span. This allows to model the external fluctuations as a white noise (see Ref. [92], pp. 101, 102). In our ecosystem the environmental fluctuations occur over time scales, ranging from some seconds to few minutes, which are much shorter than the generation time of biomass and nutrient [93]. This indicates that the condition of “rapidly fluctuating variables” is ensured, and as a consequence environmental random variations can be modelled by white noise sources.

On this basis we conclude that the specific problem can be treated by performing the integration of the stochastic differential equations within the Ito scheme.

In particular we obtain, for different values of the noise intensities, the concentration profiles averaged over 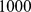 realizations [14], [94]. The presence of noise sources does not determine significant variations in the time necessary to reach the steady state. Therefore, accordingly to the deterministic analysis, in order to get the stationary solution, we solve the equations of the stochastic model fixing as a maximum time 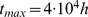.

**Figure 7:**
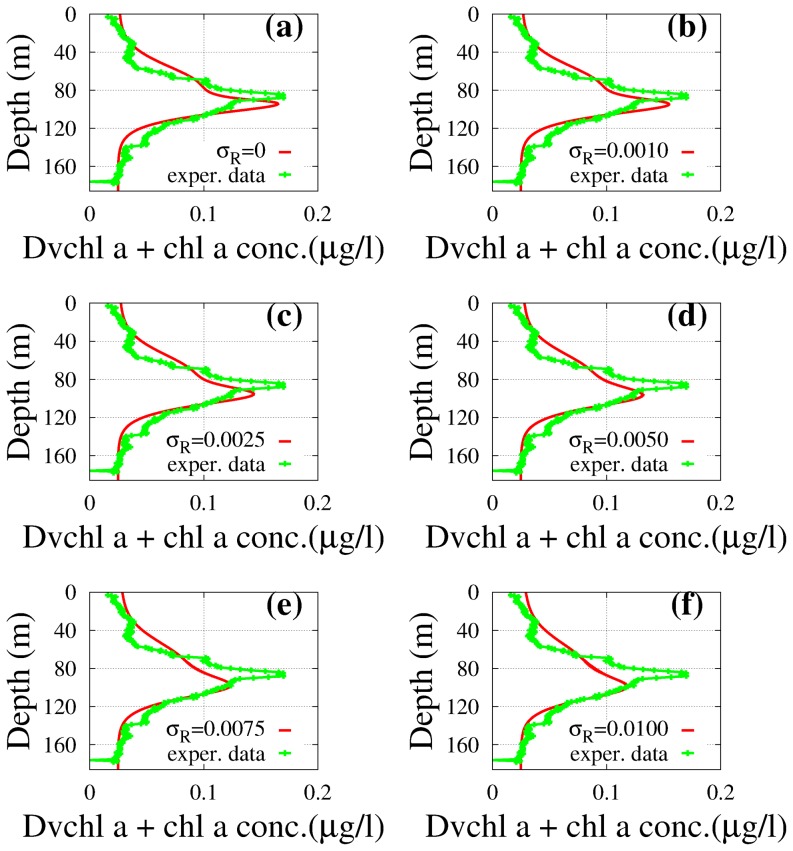
Theoretical distributions (red line) of the total *chl a* and *Dvchl a* concentration (stochastic approach). The profiles were obtained in stationary regime for different values of 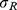 (case 1 of the stochastic model) as a function of depth. The results are compared with the distributions of the total *chl a* and *Dvchl a* concentration measured (green line) in site L1129b. The theoretical values were obtained averaging over 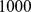 numerical realizations. The values of the parameters are those shown in Table 1. The noise intensities are: (a) 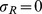 (deterministic case), (b) 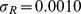, (c) 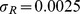, (d) 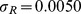, (e) 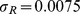 and (f) 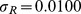. (Panels a and f: courtesy of Denaro et al., in press (Ref. [100])). (Color online).

**Figure 8:**
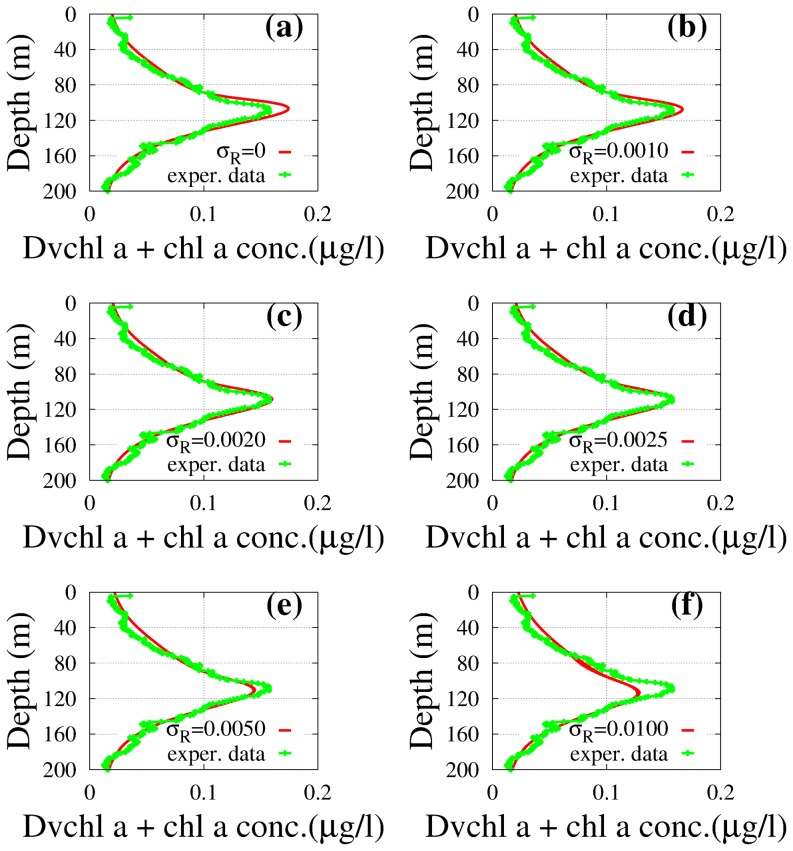
Theoretical distributions (red line) of the total *chl a* and *Dvchl a* concentration (stochastic approach). The profiles were obtained in stationary regime for different values of 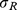 (case 1 of the stochastic model) as a function of depth. The results are compared with the distributions of the total *chl a* and *Dvchl a* concentration measured (green line) in site L1105. The theoretical values were obtained averaging over 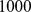 numerical realizations. The values of the parameters are those shown in Table 1. The noise intensities are: (a) 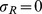 (deterministic case), (b) 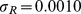, (c) 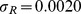, (d) 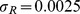, (e) 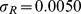 and (f) 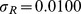. (Color online).

**Case 1.** We get the average theoretical distributions of total *chl a* and *Dvchl a* concentration in each site (see Figs. 7 and 8), by following the same procedure as in deterministic approach.

Here, the results show that a decrease and a deeper localization of the DCMs, respect to the deterministic case, are present also for low noise intensities (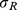 between 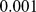 and 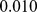). In order to evaluate the agreement of each theoretical distribution (red line) with the corresponding experimental one (green line), we use two comparative methods: 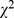 goodness-of-fit test and Kolmogorov-Smirnov (K-S) test. The results are shown in Tables 2 and 3, where 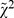 indicates the reduced chi-square, while 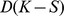 and 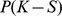 are the maximum difference between the cumulative distributions and the corresponding probability for the K-S test, respectively. The black lines have been obtained by connecting the experimental points corresponding to samples distanced of 1 meter along the water column. The quantitative comparison, based on the 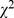 goodness-of-fit test, shows a good agreement between theoretical and experimental profiles for both sites, better than in the deterministic case. In particular, the best value of the 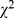 test is obtained for site L1129b with 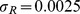, and for site L1105 with two different values of the noise intensity, i.e. 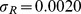 and 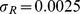. Analyzing the results of the Kolmogorov-Smirnov test we get, in site L1105, the best agreement between experimental and theoretical distributions with 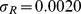, while in site L1129b the parameters 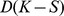 and 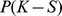 remain unchanged as 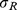 varies. We note that the best agreement in site L1105 is obtained for a value of the noise intensity 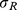 lower than that used in site L1129b. This can be explained by the fact that in site L1105 the DCM is deeper than in site L1129b (111 m vs. 88 m). As a consequence, in site L1105 the environmental variables, and therefore the peak of total *chl a* and *Dvchl a* concentration, are subject to less intense random perturbations respect to site L1129b, which is closer to the water surface.

**Table 2:** Results of 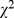, reduced chi-square (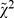), and Kolmogorov-Smirnov goodness-of-fit tests for site L1129b with different values of 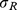 (stochastic dynamics - case 1).

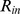	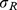	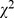	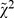	D (K-S)	P (K-S)
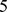	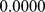	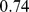	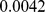	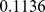	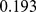
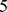	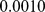	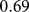	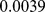	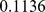	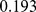
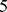	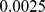	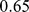	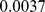	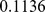	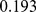
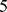	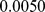	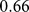	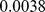	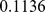	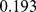
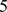	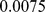	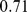	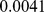	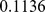	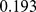
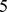	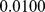	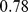	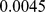	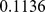	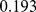

D(K-S) and P(K-S) are the maximum difference between the cumulative distributions and the corresponding probability for the K-S test, respectively. The number of samples, used for the tests and distanced of 1 m, is n = 176, corresponding to consider the whole water column.

**Table 3:** Results of 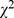, reduced chi-square (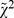), and Kolmogorov-Smirnov goodness-of-fit tests for site L1105 at different values of 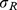 (stochastic dynamics - case 1).

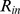	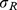	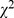	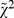	D (K-S)	P (K-S)
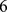	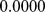	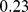	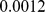	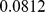	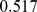
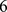	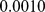	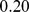	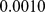	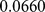	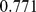
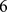	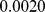	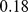	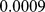	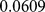	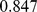
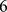	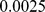	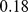	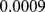	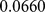	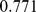
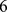	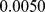	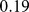	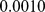	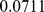	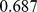
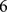	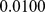	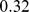	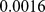	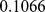	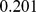

D(K-S) and P(K-S) are the maximum difference between the cumulative distributions and the corresponding probability for the K-S test, respectively. The number of samples, used for the tests and distanced of 1 m, is n = 200, corresponding to consider from the surface the first 200 m of depth.

**Figure 9:**
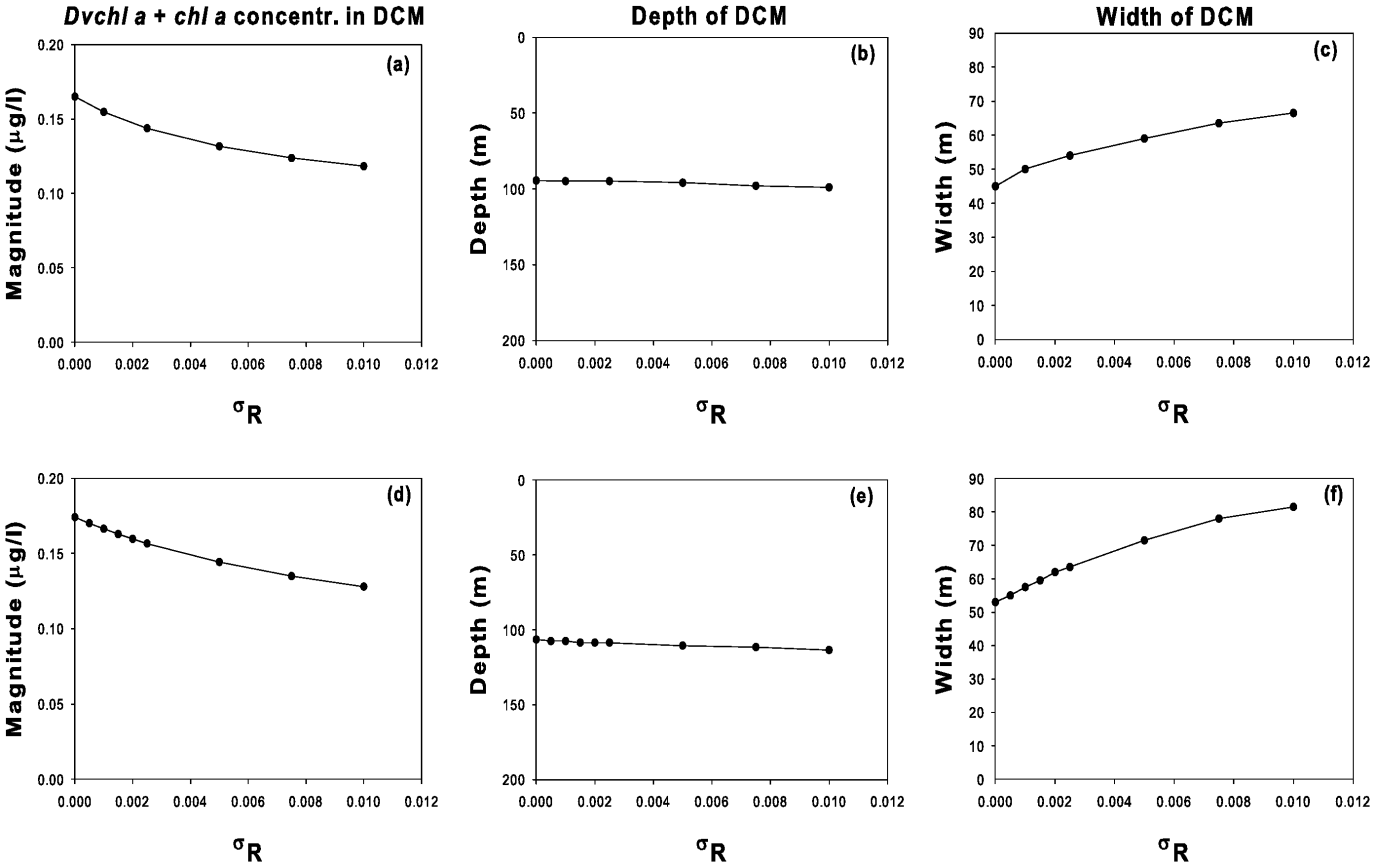
Magnitude, depth, and width of the DCM as a function of 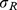 obtained from the model. The values were obtained in stationary regime for site L1129b (panels a, b, c) and site L1105 (panels d, e, f).

In order to better understand the dependence of the biomass concentration on the random fluctuations of the nutrient, we study for both sites the behaviour of the depth, width, and magnitude of the DCM as a function of 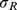. The results, shown in Fig. 9, indicate that the depth of the DCM slightly increases in both sites as a function of the noise intensity (see panels b, e). We note also that a decrease of the total concentration of *chl a* and *Dvchl a* is observed in the DCMs of the two sites (see panels a, d). At the same time we observe an increase, slightly faster in site L1105, of the width of the DCM (see panels c, f). The spread of DCM and reduction of its magnitude appear therefore to be strictly connected with each other. In general, the results (results here not shown) indicate that the phytoplankton biomass tends to disappear for 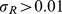. On the basis of this analysis, the nutrient concentration appears to play a crucial role in the stability of both phytoplankton groups, i.e. picoeukaryotes and Prochlorococcus. The presence of noise sources directly acting on the nutrient concentration could explain, in real ecosystems, events as the disappearance of the picophytoplankton biomass.

**Figure 10:**
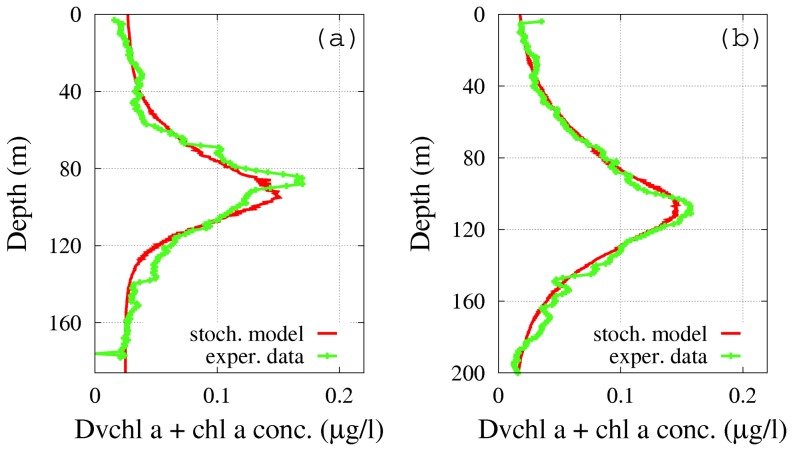
Theoretical distributions (red line) of the total *chl a* and *Dvchl a* concentration (stochastic approach). The profiles were obtained in stationary regime for a given set of noise intensities (case 2 of the stochastic model) as a function of depth, and are compared with the corresponding experimental distributions (green line) in sites L1129b (panel a) and L1105 (panel b). The theoretical values were obtained averaging over 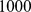 numerical realizations. The values of the parameters are those shown in Table 1. The noise intensities are: (a) 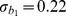, 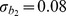 and 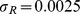 for site L1129b; (b) 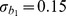, 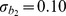 and 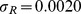 for site L1105. (Color online).

**Case 2.** According to the procedure followed for case 1, we obtain in both sites the profiles of the total concentration of *chl a* and *Dvchl a* for suitable values of the noise intensity (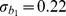, 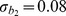 and 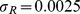 for site L1129b; 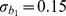, 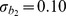 and 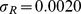 for site L1105). The results are shown in Fig. 10. In this case, the 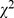 goodness-of-fit test (see Table 4) exhibits values of the reduced chi-square (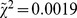 for site L1129b and 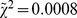 for site L1105) much lower than the values previously obtained from the stochastic approach in case 1. Viceversa, the statistical parameters, 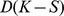 and 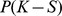, of the Kolmogorov-Smirnov test remain unchanged for site L1129b, while indicate in site L1105 a worse agreement, with respect to case 1, between numerical results and experimental data. On the basis of these results we can conclude that in site L1129b the presence of noise sources, which act on the phytoplankton biomass, allows to further improve the agreement between theoretical results and experimental findings. Contrasting indications are provided, in site L1105, by the 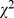 and K-S tests, about the role played by the noise sources 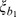 and 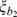 from the point of view of a better agreement between theoretical and experimental distributions.

**Table 4:** Results of 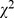, reduced chi-square (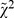), and Kolmogorov-Smirnov goodness-of-fit tests for sites L1129b and L1105, at fixed values of 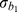, 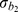 and 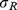 (stochastic dynamics - case 2).

Site	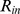	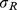	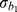	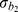	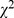	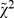	D (K-S)	P (K-S)
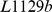	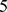	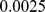	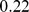	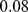	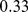	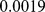	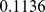	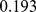
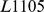	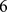	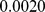	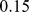	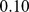	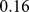	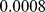	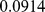	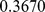

D(K-S) and P(K-S) are the maximum difference between the cumulative distributions and the corresponding probability for the K-S test, respectively. The number of samples, used for the tests and distanced of 1 m, is n = 176 for site L1129b, corresponding to consider the whole water column, and n = 200 for site L1105, corresponding to consider from the surface the first 200 m of depth.

In conclusion, the results obtained from the stochastic model indicate that the environmental fluctuations, connected with the random modifications of physical variables, such as temperature and salinity, can give rise to interesting effects: (i) “shift” of DCM towards a greater depth; (ii) “disappearance” of picoeukaryotes and Prochlorococcus for higher noise intensity. These results could explain the time evolution of picophytoplankton populations in real ecosystems whose dynamics is continuously influenced by random fluctuations of the environmental variables [7]–[9].

## Discussion

The work presented in this paper consisted in studying the dynamics of two picophytoplankton groups by using a stochastic model [27], [28], [70], [73] and comparing the results with real data from the southern Mediterranean Sea. In particular we investigated two sites of the Strait of Sicily, where the waters are prevalently oligotrophic, i.e. with low nutrient concentrations, and the climatic parameters are those typical of a temperate region. The phytoplankton groups analyzed are picoeukaryotes and Prochlococcus, which account about for 60% of total chlorophyll on average in Mediterranean Sea and belong to the smaller size fraction (less than 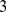 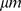) of phytoplankton, i.e. picophytoplankton.

In general, the composition of phytoplankton changes along the water column between the surface and the seabed. This is due to the fact that different groups of picophytoplankton show different responses to the limiting factors, i.e. light intensity and nutrient concentration [6]. In particular, picoeukaryotes is a nutrient-limited group localized in deep chlorophyll maximum, viceversa Prochlococcus is a light-limited species, which is forced to live close to the light source. It is important to underline that, for larger values of depth, the light intensity becomes a main limiting factor for other species of picophytoplankton, such as Synechococcus, which show a low degree of adaptability to smaller values of light intensity [61], [63] and, as a consequence, can not survive in deep layers of water column corresponding to DCM. For these reasons, we chose to analyze only the behaviour of Prochlorococcus and picoeukaryotes, which are characterized by a high degree of genetic plasticity [95] and a good adaptability to lower light intensities [55], [63], [96]. These two factors allow Prochlorococcus and picoeukaryotes to dominate in the deep chlorophyll maximum [51].

In this article, the competition between picoeukaryotes and Prochlorococcus for light and phosphorus sources has been modeled by using a advection-reaction-diffusion model [50], [97]. Moreover, the values of the biological parameters, such as maximum specific growth rates and sinking velocities, are those of picoeukaryotes and Prochlorococcus, while some environmental parameters, such as incident light intensity and depth of the water column, are fixed at values obtained from real data. Finally, for the vertical turbulent diffusivity, 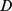, and nutrient concentration at the bottom of the water column, 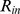, we chose values suitable to reproduce, in stationary conditions, the deep chlorophyll maximum [4], [50]. Both these parameters contribute to determine the nutrient availability along the water column and, as a consequence, to change the position, shape and magnitude of the peak of phytoplankton biomass [50]. Preliminary analysis showed that an increase of 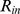 favors the better light competitor, i.e. picoeukaryotes, viceversa an increase of 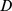 favors the better nutrient competitor, i.e. Prochlorococcus. These results are in agreement with previous works of other authors [50], [67].

In our model, we set in both sites the condition 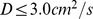, corresponding to weakly mixed waters, which causes the phytoplankton peak to have a width of some meters, as observed in the experimental data. Furthermore, we fixed low values of the nutrient concentration at the bottom (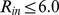 *mmol nutrient m
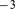
*), corresponding to the condition of oligotrophic waters in both sites. In general, for our set of parameters, the deep chlorophyll maximum is always formed by both groups, even if the peak of Prochlorococcus biomass is localized above DCM. It is important to underline that in our analysis we used along the water column a constant value of vertical turbulent diffusivity. However, in order to evaluate the effects of the presence of an upper mixed layer (UML), we analyzed the phytoplankton dynamics fixing, in the surface layers i.e. above the thermocline, a high value of vertical turbulent diffusivity (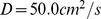). The numerical results (here not shown) were not in agreement with the experimental data. In conclusion, we observed that the approach used in Ref. [50], [67] describes the mechanisms of our ecosystem better than the Yoshiyama approach [4], [98].

In order to compare the numerical results with the experimental ones, we converted the theoretical biomass concentrations obtained from the model into *chl a* and *Dvchl a* concentration by using the mean vertical profile curves of Brunet at al. [51].

In our analysis, as a first step, we exploited a deterministic model. The results obtained show a qualitative agreement with the field observations, even if the theoretical and experimental distributions of total *chl a* and *Dvchl a* concentration present some differences. In particular, the shape of the numerical distribution of total *chl a* and *Dvchl a* concentration resulted quite different from the experimental profile in site L1129b, while the magnitude of the theoretical DCM in site L1105 was quite higher than the experimental value.

In order to take into account the effects of the noisy behaviour of the environmental variables, we inserted the contribution of the random fluctuations by adding a term of multiplicative Gaussian noise in the differential equation for the nutrient concentration (case 1). The numerical results showed that the presence of a noise source, which acts directly on the dynamics of the nutrient, allows to reproduce, in stationary conditions and for both marine sites analyzed, average profiles of the total *chl a* and *Dvchl a* concentration in a better agreement with the experimental findings respect to the deterministic case. In particular, on the basis of two comparative methods (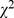 goodness-of-fit test and Kolmogorov-Smirnov test), we found that position, shape and magnitude of the DCMs agree very well with the experimental ones in both sites. Afterwards we modified the model, considering also the effects of two multiplicative Gaussian noise sources, which act directly on the two picophytoplankton groups (case 2). In these conditions, for suitable noise intensities, the 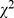 goodness-of-fit test exhibit in both sites values much lower than those previously obtained by the stochastic model in case 1. Viceversa, the values obtained from the Kolmogorov-Smirnov test became worse respect to the deterministic model in one of the two marine sites analyzed, but remained unaltered for the other site, indicating that the random fluctuations which affect the nutrient dynamics play a main role in the dynamics of the ecosystem.

### Conclusions

The results presented show that the stochastic model, which considers the dynamics of picoeukaryotes and Prochlorococcus, is able to reproduce biomass distributions in a marine ecosystem characterized by weakly mixed waters. In particular this work presents two novelties. First, a stochastic approach is used to describe the dynamics of two picophytoplankton populations. Second, theoretical results for biomass concentrations are converted into the corresponding chlorophyll content. This allows to perform a direct comparison between the chlorophyll concentration obtained by the model and the same quantity sampled in two different marine sites. A good agreement between theoretical results and experimental findings is obtained thanks to the presence of both phytoplankton groups considered in our analysis. More specifically, the approach used in this work allows to get distributions of total *chl a* and *Dvchl a* concentration in a good agreement with the experimental ones, even if the equations do not include explicitly environmental variables such as salinity, temperature and velocity field. We conclude observing that the results of this work could contribute, within the context of aquatic ecosystems, to devise a new class of models based on a stochastic approach and able to predict future changes, produced by global warming, in phytoplankton distributions.

## References

[1] Mann KH, Lazier JRN (1996) Dynamics of marine ecosystems. Malden, MA, USA: Blackwell Publishing.

[2] Klausmeier CA, Litchman E (2001) Algal games: the vertical distribution of phytoplankton in poorly mixed water columns. Limnol Oceanogr 46: 1998–2007.

[3] Huisman J, Thi NPT, Karl DM, Sommeijer B (2006) Reduced mixing generates oscillations and chaos in the oceanic deep chlorophyll maximum. Nature 439: 322–325. 16421570 10.1038/nature04245

[4] Ryabov AB, Rudolf L, Blasius B (2010) Vertical distribution and composition of phytoplankton under the influence of an upper mixed layer. J Theor Biol 263: 120–133. 19896955 10.1016/j.jtbi.2009.10.034

[5] Hickman A, Dutkiewicz S, Williams R, Follows M (2010) Modelling the effects of chromatic adaptation on phytoplankton community structure in the oligotrophic ocean. Mar Ecol Prog Ser 406: 1–17.

[6] Beversdorf L, Miller T, McMahon K (2013) The role of nitrogen fixation in cyanobacterial bloom toxicity in a temperate, eutrophic lake. PLoS ONE 8(2): e56103. 23405255 10.1371/journal.pone.0056103 PMC3566065

[7] Grenfell BT, Wilson K, Finkenstädt BF, Coulson TN, Murray S, et al (1998) Noise and determinism in synchronized sheep dynamics. Nature 394: 674–677.

[8] Zimmer C (1999) Life after chaos. Science 284: 83–86.

[9] Bjørnstad ON, Grenfell BT (2001) Noisy clockwork: Time series analysis of population fluctuations in animals. Science 293: 638–643. 11474099 10.1126/science.1062226

[10] Spagnolo B, Cirone M, La Barbera A, de Pasquale F (2002) Noise induced effects in population dynamics. J Phys 14: 2247–2255.

[11] La Barbera A, Spagnolo B (2002) Spatio-temporal patterns in population dynamics. Physica A 314: 120–124.

[12] Spagnolo B, La Barbera A (2002) Role of the noise on the transient dynamics of an ecosystem of interacting species. Physica A 315: 114–124.

[13] Spagnolo B, Fiasconaro A, Valenti D (2003) Noise induced phenomena in Lotka-Volterra systems. Fluct Noise Lett 3: L177–L185.

[14] Spagnolo B, Valenti D, Fiasconaro A (2004) Noise in ecosystems: A short review. Math Biosci Eng 1: 185–211. 20369967 10.3934/mbe.2004.1.185

[15] Valenti D, a Fiasconaro, Spagnolo B (2004) Pattern formation and spatial correlation induced by the noise in two competing species. Acta Phys Pol B 35: 1481–1489.

[16] Spagnolo B, Valenti D, Fiasconaro A (2005) Transient behavior of a population dynamical model. Prog Theor Phys Supp 157: 312–316.

[17] Caruso A, Gargano ME, Valenti D, Fiasconaro A, Spagnolo B (2005) Cyclic fluctuations, climatic changes and role of noise in planktonic foraminifera in the Mediterranean Sea. Fluct Noise Lett 5: L349–L355.

[18] Chichigina O, Valenti D, Spagnolo B (2005) A simple noise model with memory for biological systems. Fluct Noise Lett 5: L243–L250.

[19] Fiasconaro A, Valenti D, Spagnolo B (2006) Asymptotic regime in N random interacting species. Eur Phys J B 50: 189–194.

[20] Valenti D, Schimansky-Geier L, Sailer X, Spagnolo B (2006) Moment equations for a spatially extended system of two competing species. Eur Phys J B 50: 199–203.

[21] Chichigina OA (2008) Noise with memory as a model of lemming cycles. Eur Phys J B 65: 347–352.

[22] La Cognata A, Valenti D, Dubkov A, Spagnolo B (2010) Dynamics of two competing species in the presence of levy noise sources. Phys Rev E 81: 011121 (1–8).. 10.1103/PhysRevE.82.011121 20866579

[23] Chichigina OA, Dubkov AA, Valenti D, Spagnolo B (2011) Stability in a system subject to noise with regulated periodicity. Phys Rev E 84: 021134 (1–10).. 10.1103/PhysRevE.84.021134 21928976

[24] Goryachev A, Toh DJ, Wee KB, Lee T, Zhang HB, et al (2005) Transition to quorum sensing in an agrobacterium population: A stochastic model. PLoS Comput Biol 1(4): e37. 16170413 10.1371/journal.pcbi.0010037 PMC1214540

[25] Maye A, Hsieh CH, Sugihara G, Brembs B (2007) Order in spontaneous behavior. PLoS ONE 2(5): e443. 17505542 10.1371/journal.pone.0000443 PMC1865389

[26] Valenti D, Spagnolo B, Bonanno G (2007) Hitting time distributions in financial markets. Physica A 382: 311–320.

[27] Valenti D, Denaro G, La Cognata A, Spagnolo B, Bonanno A, et al (2012) Picophytoplankton dynamics in noisy marine environment. Acta Phys Pol B 43: 1227–1240.

[28] Denaro G, Valenti D, La Cognata A, Spagnolo B, Bonanno A, et al (2013) Spatio-temporal behaviour of the deep chlorophyll maximum in mediterranean sea: Development of a stochastic model for picophytoplankton dynamics. Ecol Complex 13: 21–34.

[29] Veldhuis MJW, Timmermans KR, Croot P, Van Der Wagt B (2005) Picophytoplankton; a comparative study of their biochemical composition and photosynthetic properties. J Sea Res 53: 7–24.

[30] Karsenti E, Acinas S, Bork P, Bowler C, Vargas CD, et al (2011) A holistic approach to marine eco-systems biology. PLoS Biol 9(10): e1001177. 22028628 10.1371/journal.pbio.1001177 PMC3196472

[31] Li WKW (1995) Composition of ultraphytoplankton in the central North Atlantic. Mar Ecol Prog Ser 122: 1–8.

[32] Estrada M (1996) Primary production in the North-Western Mediterranean. Sci Mar 60: 5564.

[33] Mei ZP, Finkel ZV, Irwin AJ (2009) Light and nutrient availability affect the size-scaling of growth in phytoplankton. J Theor Bio 259: 582–588. 19409906 10.1016/j.jtbi.2009.04.018

[34] Patti B, Guisande C, Bonanno A, Basilone G, Cuttitta A, et al.. (2010) Role of physical forcings and nutrient availability on the control of satellite-based chlorophyll a concentration in the coastal upwelling area of the Sicilian Channel. Sci Mar 74(3).

[35] Behrenfeld M, O’Malley R, Siegel D, McClain C, Sarmiento J, et al.. (2006) Climate-driven trends in contemporary ocean productivity. Nature 444. 10.1038/nature05317 17151666

[36] Barale V, Jaquet JM, Ndiaye M (2008) Algal blooming patterns and anomalies in the Mediter-ranean Sea as derived from the SeaWiFS data set (1998–2003). Remote Sens Environ 112: 3300–3313.

[37] Cuttitta A, Carini V, Patti B, Bonanno A, Basilone G, et al (2003) Anchovy egg and larval distribution in relation to biological and physical oceanography in the Strait of Sicily. Hydrobiologia 503: 117–120.

[38] Weston K, Fernand L, Mills DK, Delahunty R, Brown J (2005) Primary production in the deep chlorophyll maximum of the central North Sea. J Plankton Res 27: 909–922.

[39] Melbourne-Thomas J, Constable A, Wotherspoon S, Raymond B (2013) Testing paradigms of ecosystem change under climate warming in antarctica. PLoS ONE 8(2): e55093. 23405116 10.1371/journal.pone.0055093 PMC3566216

[40] Basilone G, Guisande C, Patti B, Mazzola S, Cuttitta A, et al (2004) Linking habitat conditions and growth in the european anchovy (Engraulis encrasicolus). Fish Res 68: 9–19.

[41] Klausmeier CA, Litchman E, Levin SA (2007) A model of flexible uptake of two essential resources. J Theor Biol 246: 278–289. 17291537 10.1016/j.jtbi.2006.12.032

[42] Thingstad TF, Rassoulzadegan F (1995) Nutrient limitations, microbial food webs, and biological C-pumps: suggested interactions in a P-limited Mediterranean. Mar Ecol Prog Ser 117: 299306.

[43] Ribera d’Alcalµa M, Civitarese G, Conversano F, Lavezza R (2003) Nutrient ratios and fluxes hint at overlooked processes in the Mediterranean Sea. J Geophys Res 108.

[44] Norberg J (2004) Biodiversity and ecosystem functioning: a complex adaptive systems approach. Limnol Oceanogr 49: 1269–1277.

[45] Yeo SK, Huggett M, Eiler A, Rappè M (2013) Coastal bacterioplankton community dynamics in response to a natural disturbance. PLoS ONE 8(2): e56207. 23409156 10.1371/journal.pone.0056207 PMC3567041

[46] Fogg GE (1991) The phytoplanktonic ways of life. New Phytol 118: 191–232. 10.1111/j.1469-8137.1991.tb00974.x 33874179

[47] Prézelin BB, Tilzer MM, Schofield O, Haese C (1991) The control of the production process of phytoplankton by the physical structure of the aquatic environment with special reference to its optical properties. Aquat Sci 53: 136–186.

[48] Brunet C, Casotti R, Vantrepotte V, Corato F, Conversano F (2006) Picophytoplankton diversity and photoacclimation in the Strait of Sicily (Mediterranean Sea) in summer. I. Mesoscale variations. Aquat Microb Ecol 44: 127–141.

[49] Garczarek L, Dufresne A, Rousvoal S, West NJ, Mazard S, et al (2007) High vertical and low horizontal diversity of prochlorococcus ecotypes in the Mediterranean Sea in summer. FEMS Microbiol Ecol 60: 189–206. 17391326 10.1111/j.1574-6941.2007.00297.x

[50] Ryabov A (2012) Phytoplankton competition in deep biomass maximum. Theor Ecol 5: 373–385.

[51] Brunet C, Casotti R, Vantrepotte V, Conversano F (2007) Vertical variability and diel dynamics of picophytoplankton in the Strait of Sicily, Mediterranean Sea, in summer. Mar Ecol Prog Ser 346: 15–26.

[52] Casotti R, Brunet C, Aronne B, Ribera d’Alcalà M (2000) Mesoscale features of phytoplankton and planktonic bacteria in a coastal area as induced by external water masses. Mar Ecol Prog Ser 195: 15–27.

[53] Casotti R, Landolfi A, Brunet C, D’Ortenzio F, Mangoni O, et al.. (2003) Composition and dynamics of the phytoplankton of the Ionian Sea (Eastern Mediterranean). J Geophys Res 108.

[54] Raven JA, Finkel ZV, Irwin AJ (2005) Picophytoplankton: bottom-up an top-down controls on ecology and evolution. J Geophys Res 55: 209–205.

[55] Dimier C, Corato F, Saviello G, Brunet C (2007) Photophysiological properties of the marine picoeukaryotes picochlorum rcc 237 (Trebouxiophyceae chlorophyta). J Phycol 43: 275283.

[56] Worden AZ, Not F (2008) Microbial Ecology of the Oceans, Second Edition. John Wiley & Sons, Inc.

[57] Mendonça A, Arístegui J, Vilas J, Montero M, Ojeda A, et al (2012) Is there a seamount effect on microbial community structure and biomass? The case study of Seine and Sedlo Seamounts (Northeast Atlantic). PLoS ONE 7(1): e29526. 22279538 10.1371/journal.pone.0029526 PMC3261146

[58] Raven JA (1998) The twelfth tansley lecture. small is beautiful: The picophytoplankton. Funct Ecol 12: 503.

[59] Brunet C, Casotti R, Aronne B, Vantrepotte V (2003) Measured photophysiological parameters used as tools to estimate vertical water movements in the coastal Mediterranean. J Plankton Res 25: 1413–1425.

[60] Finkel ZV, Irwin AJ (2005) Picophytoplankton: Bottom-up and top-down controls on ecology and evolution. Vie Milieu 55: 209–215.

[61] Brunet C, Casotti R, Vantrepotte V (2008) Phytoplankton diel and vertical variability in photo-biological responses at a coastal station in the Mediterranean Sea. J Plankton Res 30: 645–654.

[62] Dimier C, Saviello G, Tramontano F, Brunet C (2009) Comparative ecophysiology of the xantho-phyll cycle in six marine phytoplanktonic species. Protist 160: 397–411. 19375387 10.1016/j.protis.2009.03.001

[63] Moore LR, Goericke R, Chisholm SW (1995) Comparative physiology of synechococcus and prochlorococcus: influence of light and temperature on growth, pigments, fluorescence and absorptive properties. Mar Ecol Prog Ser 116: 259–275.

[64] Partensky F, Hess W, Vaulot D (1999) Prochlorococcus, a marine photosyntheic prokaryote of global significance. Microbiol Mol Biol R 63: 106–127. 10.1128/mmbr.63.1.106-127.1999 PMC98958 10066832

[65] Bougaran G, Bernard O, Sciandra A (2010) Modeling continuous cultures of microalgae colimited by nitrogen and phosphorus. J Theor Biol 265: 443454. 10.1016/j.jtbi.2010.04.018 20433853

[66] Turpin DH (1988) Physiological mechanisms in phytoplankton resource competition (316–368). In: Growth and reproductive strategies of freshwater phytoplankton. Cambridge University Press.

[67] Ryabov A, Blasius B (2011) A graphical theory of competition on spatial resource gradients. Ecol Lett 14: 220–228. 21265973 10.1111/j.1461-0248.2010.01574.x

[68] Shigesada N, Okubo A (1981) Effects of competition and shading in planktonic communities. J Math Biol 12: 311–326.

[69] Kirk JTO (1994) Light and Photosynthesis in Aquatic Ecosystems (2nd edition). Cambridge University Press.

[70] Valenti D, Fiasconaro A, Spagnolo B (2004) Stochastic resonance and noise delayed extinction in a model of two competing species. Physica A 331: 477–486.

[71] Huppert A, Blasius B, Olinkya R, Stone L (2005) A model for seasonal phytoplankton blooms. J Theor Biol 236: 276–290. 15916773 10.1016/j.jtbi.2005.03.012

[72] Ebeling W, Spagnolo B (2005) Noise in condensed matter and complex systems. Fluct Noise Lett 5: L159–L161.

[73] Liu QX, Jin Z, Li BL (2008) Resonance and frequency-locking phenomena in spatially extended phytoplankton-zooplankton system with additive noise and periodic forces. J Stat Mech-Theory E 2008.

[74] Provata A, Sokolov I, Spagnolo B (2008) Editorial: Ecological complex systems. Eur Phys J B 65: 307–314.

[75] Spagnolo B, Dubkov AA (2008) Editorial of critical phenomena and diffusion in complex systems. Int J Bifurcat Chaos 18: 2643–2647.

[76] Valenti D, Tranchina L, Cosentino C, Brai M, Caruso A, et al (2008) Environmental metal pollution considered as noise: Effects on the spatial distribution of benthic foraminifera in two coastal marine areas of Sicily (Southern Italy). Ecol Model 213: 449–462.

[77] Spagnolo B, Spezia S, Curcio L, Pizzolato N, Fiasconaro A, et al (2009) Noise effects in two different biological systems. Eur Phys J B 69: 133–146.

[78] Mikhailov AS, Loskutov AY (1996) Foundations of Synergetics II: Chaos and Noise. Springer Series in Synergetics. Springer, Berlin.

[79] Muñoz MA, Colaiori F, Castellano C (2005) Mean-field limit of systems with multiplicative noise. Phys Rev E 72: 056102. 10.1103/PhysRevE.72.056102 16383683

[80] Manor A, Shnerb N (2009) Multiplicative noise and second order phase transitions. Phys Rev Lett 103: 030601. 19659259 10.1103/PhysRevLett.103.030601

[81] Schenzle A, Brand H (1979) Multiplicative stochastic processes in statistical physics. Phys Rev A 20: 1628–1647.

[82] Graham R, Schenzle A (1982) Carleman imbedding of multiplicative stochastic processes. Phys Rev A 25: 1731–1754.

[83] García-Ojalvo J, Sancho JM (1999) Noise in Spatially Extended Systems. Springer, New York. 10.1103/physreve.49.2769 9961542

[84] Redner S (1990) Random multiplicative processes: An elementary tutorial. Am J Phys 58: 267–273.

[85] Sornette D (2000) Critical Phenomena in Natural Sciences. Springer Series in Synergetics. Springer, Heidelberg.

[86] Barrera P, Ciuchi S, Spagnolo B (1993) Generating function for a multiplicative noise with group analysis. J Phys A: Math Gen 26: L559–L565.

[87] Ciuchi S, de Pasquale F, Spagnolo B (1993) Nonlinear relaxation in the presence of an absorbing barrier. Phys Rev E 47: 3915–3926. 10.1103/physreve.47.3915 9960464

[88] Ciuchi S, de Pasquale F, Spagnolo B (1996) Self-regulation mechanism of an ecosystem in a non-gaussian fluctuation regime. Phys Rev E 54: 706–716. 10.1103/physreve.54.706 9965118

[89] Dimier C, Brunet C, Geider R, Raven J (2009) Growth and photoregulation dynamics of the picoeukaryote Pelagomonas calceolata in fluctuating light. Limnol Oceanogr 54: 823–836.

[90] Moon-Van Der Staay SY, De Wachter R, Vaulot D (2001) Oceanic 18S rDNA sequences from picoplankton reveal unsuspected eukaryotic diversity. Nature 409: 607–610. 11214317 10.1038/35054541

[91] Fennel K, Boss E (2003) Subsurface maxima of phytoplankton and chlorophyll: Steady-state solutions from a simple model. Limnol Oceanogr 48: 1521–1534.

[92] Horsthemke W, Lefever R (1984) Noise-Induced Transitions: Theory and Applications in Physics, Chemistry, and Biology. Springer-Verlag, Berlin Heidelberg.

[93] Brunet C, Lavaud J (2010) Can the xanthophyll cycle help extract the essence of the microalgal functional response to a variable light environment? J Plankton Res 32: 1609–1617.

[94] Giuffrida A, Valenti D, Ziino G, Spagnolo B, Panebianco A (2009) A stochastic interspecific competition model to predict the behaviour of Listeria monocytogenes in the fermentation process of a traditional sicilian salami. Eur Food Res Technol 228.

[95] Bibby TS, Mary I, Nield J, Partensky F, Barber J (2003) Low light adapted prochloroccus species possess specific antennae for each photosystem. Nature 424: 1051–1054. 12944966 10.1038/nature01933

[96] Moore LR, Rocap G, Chisholm SW (1998) Physiology and molecular phylogeny of coexisting Prochlorococcus ecotypes. Nature 397: 464–467. 10.1038/30965 9624000

[97] Huisman J, Sharples J, Stroom JM, Visser PM, Kardinaal WEN, et al (2004) Changes in turbulent mixing shift competition for light between phytoplankton species. Ecology 85: 2960–2970.

[98] Yoshiyama K, Mellard J, Litchman E, Klausmeier C (2009) Phytoplankton competition for nu-trients and light in a stratified water column. Am Nat 174. 10.1086/600113 19538096

[99] Rippka R, Coursin T, Hess W, Lichtle C, Scanlan DJ, et al (2000) Prochlorococcus marinus Chisholm et al. 1992 subsp. pastoris subsp. nov. strain PCC 9511, the first axenic chlorophyll *a* _2_/*b* _2_-containing cyanobacterium (Oxyphotobacteria). Int J Syst Evol Micr 50: 1833–1847. 10.1099/00207713-50-5-1833 11034495

[100] Denaro G, Valenti D, Spagnolo B, Bonanno A, Basilone G, et al. (2013) Stochastic Dynamics of Two Picophytoplankton Populations in a Real Marine Ecosystem. Acta Phys. Pol. B. vol 44. pp. 977-990. In press. DOI:10.5506/APhysPolB.44.977

